# Meet the editorial board members: Angeline Ferdinand and Michael Murray

**DOI:** 10.1186/s44263-023-00010-9

**Published:** 2023-07-31

**Authors:** Angeline S. Ferdinand, Michael F. Murray

**Affiliations:** 1 Microbiological Diagnostic Unit Public Health Laboratory, Department of Microbiology and Immunology, University of Melbourne at the Doherty Institute, Victoria, Australia; 2 Mount Sinai Health System, New York, USA

## Abstract

In this Q&A, Angeline Ferdinand and Michael Murray answer questions about their research fields and share insights into their role as editorial board members at the journal.

## What is the focus of your research and what drew you to this field?

*Angeline S. Ferdinand*: Like many in this field, my research is hugely varied, which I enjoy. My discipline is in Evaluation and Implementation Science, and I am fortunate to be able to apply this lens to public health programs, services and policies across public health implementation of pathogen genomics, One Health approaches to antimicrobial resistance and equitable access to health services. The field of global and public health holds particular attraction because it allows me to draw on perspectives from across disciplines, sectors and settings to address issues that really matter around equity, the transformative power of technology and the interconnected nature of health and wellbeing.

*Michael F. Murray*: My research focuses on advancing the incorporation of genetics and genomics into healthcare, with the aim of improving the health in both individuals and populations. There is much work needed if we are going to be able to understand how to best use DNA-based technologies for both accurate diagnoses within healthcare and as the basis of population health screening.

In 2000, I made a career shift; at that time, I was a practicing infectious disease physician and I left practice to train further in genetics and genomics with a focus on variable host susceptibility to infectious agents. My work in genomics has since broadened beyond the focus on susceptibility to infections to include work aimed at understanding genomic risk for common diseases such as cancer and heart diseases.

## What are the main problems that need addressing in the field?

*ASF*: Technological advancements to address urgent and ongoing threats to global public health have transformed approaches to communicable and non-communicable diseases. A potential pitfall is the application of new technologies without careful consideration of appropriate integration. Disjointed introduction of new technologies can lead to challenges in sustainability and utilisation, leading to a waste of resources. In the worst case scenarios, the introduction of new technologies can cause disruption where resources are redirected from other, already stressed, parts of the health care system. In this way, technological approaches can serve to undermine public health strategies that are not technology-dependent, including those that address social determinants of health. This also has implications for health equity, which can either be supported or stymied by introduction of new health technologies.

*MFM*: There is significant enthusiasm for implementing genomic technologies in healthcare and population screening. This includes enthusiasm from many sectors beyond scientists and clinicians including governments and private corporations.

The major challenge that needs to be addressed is the expansion of our limited evidence-base for the clinical utility of DNA-based strategies to improve health. Evidence for the demonstrable utility of specific applications DNA-based technologies are necessary if we are to avoid implementation strategies that drive costs without sufficient benefits.

## How do you expect this field to develop in the next few years?

*ASF*: With the breadth of the field and enormously rapid changes the field is undergoing, this is a very difficult question to answer. Over the past few years, the important and positive trend of more open dissemination and sharing of data, research findings and ideas has led to the acceleration of innovation and an expectation of transparency in research and practice. I expect this to continue, alongside continued movement towards interdisciplinary and transdisciplinary approaches, as researchers, practitioners and policymakers respond to increasingly complex problems that necessitate integration of varied perspectives and understandings.

*MFM*: The ability of research groups to launch large scale population studies will be increased by two trends: the decreasing costs of DNA technologies, and an increasing capacity to interpret rare genetic variants. This kind of research, particularly efforts that engage diverse human populations will expand the knowledge base for the benefit of all.

## What are you most excited about in your role as an Editorial Board Member for the journal?

*ASF*: I am thrilled to be able to contribute to this new journal that aligns so well with my own interests and occupies an important space in the field. I’m particularly excited to have the opportunity to review up-to-the-minute work and engage with researchers at the forefront of addressing some of the most pressing issues in global health. As part of the Editorial Board, I am joining a team of researchers shaping policy and practice in areas as diverse as migrant and refugee health, health financing, environmental medicine, mathematical modelling and pathogen genomics. I’m looking forward to building new networks and connections as part of this role.

*MFM*: This journal can provide a platform for work focused on the implementation of genomic medicine in global and public health. My role as an Editorial Board Member will allow me for the first time in my career to contribute to the selection and advancement of manuscripts in which authors will have the opportunity to share their important international work on this new platform.

## Data Availability

Not applicable.

